# A Micro-Computed Tomography-Based Simplified Approach to Measure Body Composition, Osteoporosis, and Lung Fibrosis in Mice

**DOI:** 10.21769/BioProtoc.5207

**Published:** 2025-02-20

**Authors:** Madeleine B. Landau, Binghao Zou, Ziqi Yang, Brian G. Rowan, Muralidharan Anbalagan

**Affiliations:** 1 Tulane University School of Medicine, New Orleans, LA, USA; 2 Department of Structural and Cellular Biology, Tulane University School of Medicine, New Orleans, LA, USA

**Keywords:** Micro-computed tomography, Micro-CT, Murine models, Body composition, Bone mineral density, Adipose, Lean tissue, Lung fibrosis, Tissue structure

## Abstract

Micro-computed tomography (micro-CT) is a powerful, non-destructive imaging technique that creates high-resolution 3D images of the internal structures of small animal models such as mice and rats. Familiarizing oneself with micro-CT imaging and data analysis can be overwhelming without easy-to-follow, clear instructions. Training on new instruments is often a task exclusive to a select subset of researchers, leaving the majority of potential trainees without a technical grasp of how to navigate the instructions. This protocol on the use of micro-CT aims to bridge that gap by providing a clear, step-by-step guide to acquire and analyze micro-CT images from mice for quantitative data. By exclusively detailing the necessary procedural steps from start to finish and overcoming complex user interfaces during imaging operations and analysis, this protocol will equip new micro-CT users with the ability to measure mouse body composition (bone, body fat, and lean muscle mass) and identify and quantify lung fibrosis. This approach applies to researchers with a basic understanding of medical imaging, animal care, and software analysis.

Key features

• Analysis of tissue-specific body composition using mice as model organisms.

• An easy-to-follow guide for novice users of high-resolution micro-computed tomography imaging systems.

• Enhances accessibility, workflow, standardization, training, and breadth of application in the research community.

• Effectively employing non-invasive live imaging allows for a longitudinal study of tissue architecture for examining age-related changes in vivo.

Graphical overview

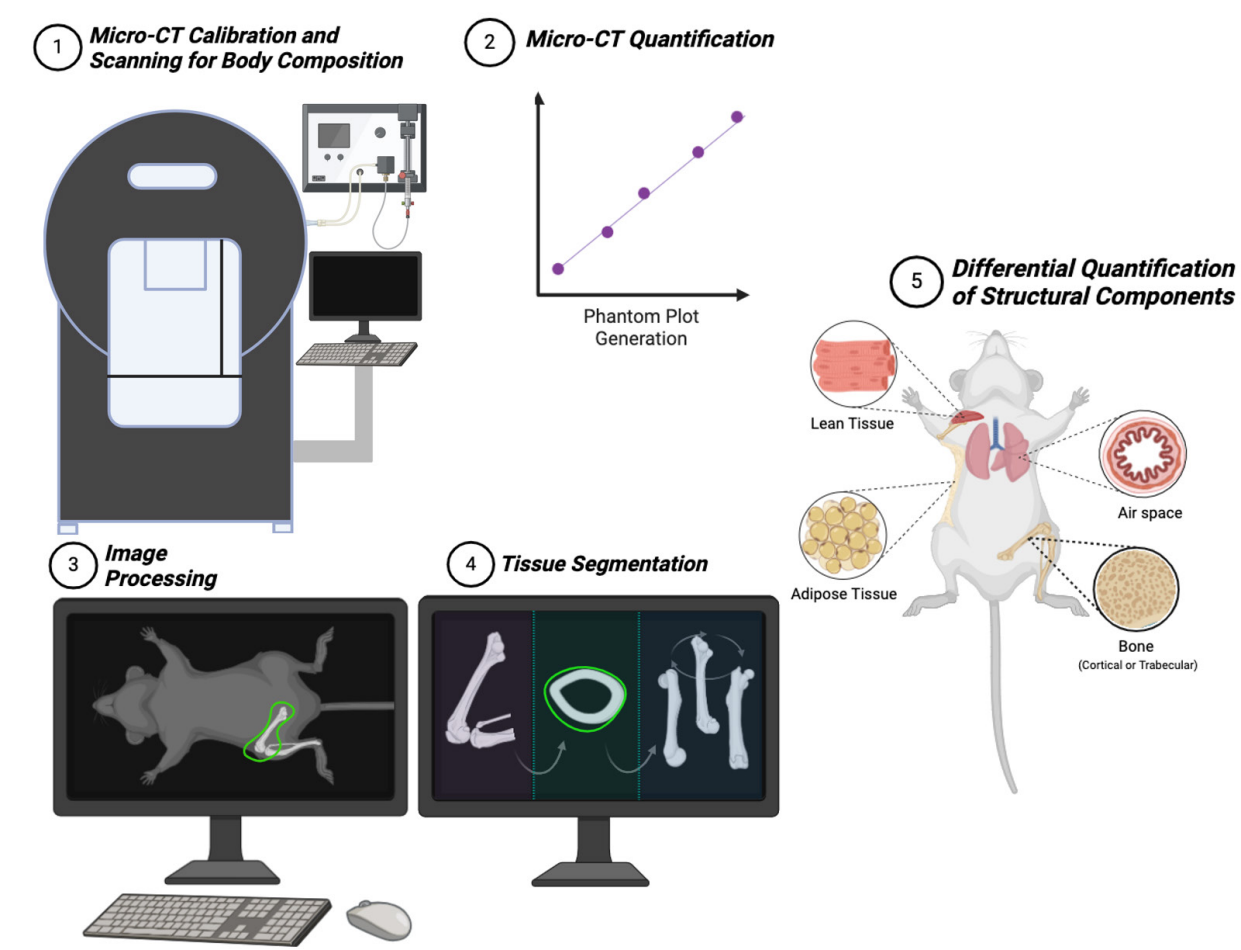

## Background

The role of small animal models in basic science research has rapidly expanded amidst the emergence of novel quantification techniques [1]. Precision measurement in such models has endeavored to unearth underlying mechanisms of action in estrogen-receptor signaling and innovative modes of drug delivery for quality improvement [2,3]. Genetically modified mice have an indispensable purpose in assessing the role of specific genes in key physiological functions and phenotypic changes that are often measured through histological analysis [4,5]. Nonetheless, such models may fail to provide either pertinent longitudinal data, an important consideration for examining structural development (fat, muscle, bone), or pathological changes in tissues such as lung fibrosis.

As an alternative approach to examining in vivo features at a fixed point in time, non-invasive techniques that allow for periodic imaging over time can quantitatively measure varied physiologic and pathologic phenotypes [5]. The benefits of live imaging of murine structures include quantifiable measurements for organic tissue-dependent changes at specific growth stages without inflicting harm or interruption to the life cycle. Non-invasive tools also enhance experimental designs by yielding a series of data points over the entire study duration, limiting the overall cohort size needed, and allowing more precise measures of inter-animal variability [6].

One of the most frequently utilized tools for diagnostic imaging is X-ray computed tomography (CT), which relies on employing X-ray beams in direct contact with the organism to create a cross-sectional tomographic representation. Hounsfield units (HU) is a relative quantitative measurement of radio density used in the interpretation of computer tomography images in the linear scale; the radio density of distilled water at a standard temperature and pressure is defined as 0 HU, whereas the radio density of air is -1,000 HU. A phantom with a known radio density calibrates machines, ensuring they provide the correct HU. Most animal tissues do not exceed +2,000 HU and standard clinical limits range from -1,000 to +3,071 [7].

In pre-clinical research, the ability of the micro-CT scanner to elicit information on body composition comes highly appreciated, enhancing surveillance of disease development, classification, and ramifications [8]. Whether analyzing bone mineral density for studying osteoporosis, distinguishing airspace changes for characterizing lung fibrosis, or comparing adipose volume change, non-invasive in vivo imaging using micro-CT scanners enables high spatial resolution with longitudinal monitoring [9].

Without a standardized protocol for utilization, not only does the reproducibility of research suffer, but it also hinders the ability to build upon the ever-evolving field of structural analysis and to develop an eventual gold standard for body composition characterization. Simple method articles serve as valuable resources for researchers who wish to incorporate micro-CT into their studies.

In this article, we propose an easy-to-follow guide for micro-CT users to reconstruct the physical structure of mice to obtain standard non-invasive imaging in mice-based long-term experiments. Our protocol improves upon existing methods by providing accessible, step-by-step instructions that minimize the need for specialized training. It simplifies workflows into manageable tasks, promotes standardization with many visual aids and troubleshooting guidance, and improves reliability. This protocol details a reproducible manner by which researchers can precisely attain data on lean tissue, adipose, bone structure, bone mineral density, and airspace quantification in the lung, enabling the investigation of gene-based translational applications.

## Materials and reagents

**Biological materials**

1. C57BL/6 mice, 3 months old (C57BL/6J from The Jackson Laboratory, Bar Harbor, ME)

**Reagents**

1. Fluriso^TM^, isoflurane (VETone, catalog number: 502017), store at 20–25 °C for up to five years

2. Oxygen [50 pounds per square inch gauge (psig) (345 kPA) to 60 psig (414 kPA) required] (air gas)

*Note: Oxygen can be a fire hazard, so always turn it off when not in use. Do not expose oxygen cylinders to temperatures higher than 50 °C. The oxygen tank should be securely fastened upright to prevent falling off and kept in a well-ventilated area completely separated from flammable materials and heating sources. Always keep the oxygen tank valve protected with the cap when not in use and ensure proper labeling indicating the contents as OXYGEN and whether it is full or empty. Never attempt to handle a damaged or leaking cylinder. Be aware of your laboratory-specific safety procedures while handling the oxygen tank.*

3. Bleomycin solution 10 mg/mL in water (Sigma, catalog number: B7216); should be protected from light and stored at -20 °C. Mice were given 60 µL of bleomycin (1.25 U·kg^-1 ^body weight) via oropharyngeal route to induce a fibrotic response that is more pronounced at day 21 after the treatment [10]. The Institutional Animal Care Use Committee (IACUC) approved this bleomycin-induced lung fibrosis protocol (Protocol ID: 1874)

## Equipment

1. RAS-4 anesthesia system (PerkinElmer, catalog number: CLS146737)

2. Quantum GX2 micro-CT (PerkinElmer, catalog number: CLS149276)

3. PC: DELL Precision 5820 tower XCTO high-performance acquisition computer, Windows^®^10 Pro, Intel^®^ (R) Xeon (R) W-2123 processor, NVIDIA Quadro P2000 5GB graphics card, 32 GB 2666 MHz RAM, 8 TB HD.

*Note: Researchers working with animals in our vivarium must have taken proper animal training and lectures. They must wear appropriate personal protective equipment (PPE), including a dedicated lab coat or disposable gown, gloves, mask, and head and shoe covers. All PPE is available at the entrances to our animal facilities. Additionally, researchers must take radiation safety training before operating the micro-CT instrument. We recommend that researchers follow the institution-dependent regulations and use PPE accordingly.*

## Software and datasets

1. Quantum GX2 Image Analysis Software (PerkinElmer)

2. PerkinElmer Database, version 3.5.3.110 (free software with the purchase of Quantum GX2)

3. Analyze 14.0 (AnalyzeDirect, Inc., Lexana, KS, USA, license needed, https://analyzedirect.com/analyze14/)

4. Prism v9.3 (GraphPad, 11/15/2021, license needed, https://www.graphpad.com/). To perform a t-test in Prism: Launch the software and create a new project. Choose the *Column* table format and enter the data into the appropriate columns. Click the *Analyze* button and, under the *Column Analyses* group, select *t-tests (and nonparametric tests)*.

5. BioRender (https://www.biorender.com/). The following figures were created using BioRender: Graphical overview, Created in BioRender. Landau, M. (2024) https://BioRender.com/c08f795.

## Procedure

**A. Micro-CT calibration and scanning for body composition**

**Figure 1.**
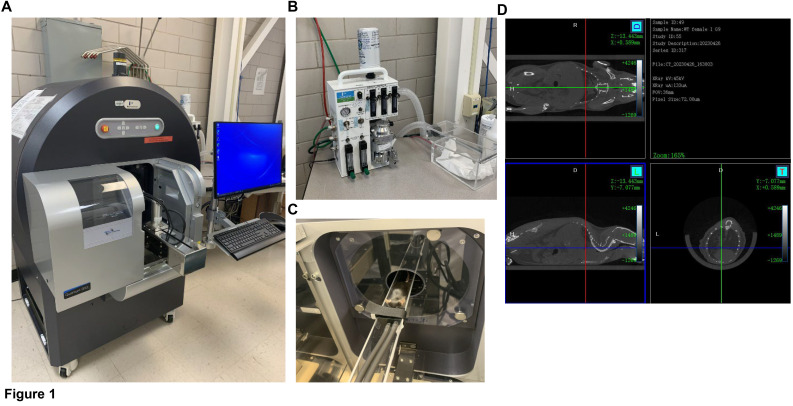
Quantum GX2 micro-CT imaging system. A) Quantum GX2 micro-CT system with the computer in our facility. B) RAS-4 rodent anesthesia system. C) Anesthetized C57BL/6 mouse placed on the mouse bed with the nose inserted inside the nose cone continuously receiving anesthesia from the RAS-4 anesthesia system. D) Mouse’s whole body scan with coronal, sagittal, and axial views.

1. Turn on the Quantum GX2 Micro-CT imaging system and switch the safety key to *On* (Figure 1A).

2. Open the Quantum GX2 Image Analysis Software and click the ***Warm-Up*** button in the lower left corner.

**Pause point:** The warm-up process takes 15 min if the duration of imaging system disuse is <1 month. For durations of 1–3 months, it takes 40 min; for >3 months, it takes 2 h.

3. **Critical:** In the database window, click ***Create New Database*** to set the folder for saving the imaging data files. Then click ***Create New Sample*** and enter the information of the new sample to set the folder for saving the data of the particular scan. Click the sample that will be scanned next and click ***Set Image Save Location***.

4. The Quantum GX2 micro-CT may require gain or HU calibration if the indicator turns yellow or red before animal scanning.

a. To complete the gain calibration, select ***Gain Calibration*** from ***Options*** of the control panel. Select the required *Scan mode, Filter*, and *Voltage* combination that is needed to be calibrated and click ***Execute*** (Figure S1A).

*Note: The indicator should turn green after the gain calibration process is complete.*

b. Set up a HU calibration water phantom by adding 25 mL of DI water into a 50 mL conical centrifuge tube. Place the water phantom into the Quantum GX2 micro-CT and configure the control panel to the required *Scan mode, Filter*, and *Voltage* combination. Create a new sample to store the scan file. Enter live mode by clicking the ***Live Mode*** button. *Stage control* on the front panel is used to adjust the position of the bed, and *rotation control* is used to confirm that the conical centrifuge tube is centered in the field. Click ***Start Scan*** to complete a scan.

c. After scanning, right-click the sample in the *series information* and select ***Launch HU Calibration***. Select ***Settings*** and ***VOX Number Calibration (V)*** to launch an ROI selection window. Move the green square that represents *AIR* to the air area in the conical centrifuge tube and the *TARGET* green square to the water area. In the *CT number adjustment*, click ***ROI read*** for AIR CT and TARGET CT and click ***OK*** (Figure S1B). Close the measurement window. On the control panel, select ***Options*** and ***HU Calibration Settings***. Find the scan mode that requires HU Calibration, choose the most recent calibration file, and the indicator should turn green (Figure S1C).

5. Before beginning animal scanning, anesthetize the mouse with 2.5% isoflurane in the RAS-4 rodent anesthesia system induction chamber.

*Note: It takes 2 min to fully anesthetize a mouse (Figure 1B).*

6. Place the anesthetized mouse on the mouse bed and close the imaging system door (Figure 1C).

**Critical:** Place only one mouse into the mouse bed at a time. Individual micro-CT scanning is encouraged for animal safety and best imaging results. The mouse's nose must be fully inserted into the nose cone to ensure consistent anesthesia delivery through inhalation of isoflurane (Figure S2).

7. Adjust the scan settings in the control window as follows:

a. **For bone marrow density (BMD):** voltage: 90 kV; current: 88 µA; acquisition: 36; recon: 36; scan mode: standard, 18 s; display settings: off; X-ray filter: Cu 0.06+Al 0.5.

b. **For body composition (other):** voltage: 45 kV; current: 133 µA; acquisition: 36; recon: 36; scan mode: standard, 18 s; display settings: off; X-ray filter: none.

8. The field of view (FOV) will present the size of each micro-CT scan. A FOV of 36 mm was used, which requires stitching five separate scans together to capture the entire mouse.

*Note: A larger FOV of 72 mm requires just three scan stitches to obtain the entire coverage. However, increasing from 36 to 72 mm for the FOV will move the camera further away from the mouse, subsequently reducing the resolution of the image. For larger rodents, such as rats or rabbits, a larger FOV may be the only plausible option.*

9. Each of the four changeable filters can be used for optimal imaging of different structures:

a. No filter (open): low contrast samples at low voltages.

b. AI 0.5 mm: Low contrast samples.

c. AI 1.0 mm: Soft tissue (fat analysis).

d. AI 0.5 mm + Cu 0.06 mm: Standard CT scanning.

e. Cu 0.1 mm: Dense samples at high voltages.

10. Set the body orientation as desired. Click ***Initialize*** in the control window to initialize the stage control.

11. At the bottom of the control window, click ***Live Mode*** to turn on the live mode and use the stage controls on the front control panel to locate the animal in the center of the live window. Use the “rotation control” to ensure the animal is centered. Move the stage to position the nose of the mouse to the center of the FOV and record the stage location.

*Note: This should be the starting point of the five stitches.*

12. To set a stitching job, select ***Options*** > ***Job Scan Settings*** and click ***New*** in the *Job Menu* (Figure S3). Press ***Add*** in the *Row Menu* to create a 5-Job scan. Enter the recorded number of the position of the nose as the starting point in the *stage Z(mm)*.

**Pause Point:** The Quantum GX2 can stitch up to five scans, which is sufficient to reconstruct the micro-CT image for the body of an adult mouse at a 36 mm FOV.

**Pause Point:** The range of the stage for stitching is 2–206, so it may be necessary to adjust the location of the nose cone to ensure that the starting and the end of the five stitches do not exceed this range.

13. Change the *stitching* to ***On*** and adjust the other parameters that are required for the scan. Click ***OK***.

14. In ***Scan Acquisition*** settings, select the job just created and confirm the parameters are correct for the scan.

15. Start scanning by clicking the CT Scan button. When the machine status becomes *stand by*, remove the animal and ensure the *X-RAY ON* light is off.

*Note: Scans are viewable from the coronal, sagittal, and axial viewpoints (Figure 1D).*

16. A phantom plot is required to calculate the standard curve for BMD quantification. A phantom has five different densities (0, 50, 200, 800, and 1,200 mg HA/cc) of hydroxyapatite (HA). Use the same setting for the phantom as for BMD scanning (Figure S4A).

**B. Micro-CT phantom plot generation**

1. To analyze 3D scans generated from the Quantum GX2 micro-CT, the associated software, the Quantum GX2 Database application, must be opened on the desktop. The user must consent to allow an existing database to connect. Once the data via USB is transferred to the computer, and upon enabling the existing database to connect to the Quantum GX2 micro-CT program, each scan becomes viewable.

**Pause Point:** If Analyze 14.0 is not installed on the same computer used to control Quantum GX2 scanning, the data must be transferred to a computer with Analyze 14.0 software. To transfer data, copy the entire database folder (the same directory as shown on the database location) or select *Export File* to export the selected sample.

2. In the Quantum GX2 Database application, select a given image file for loading, then double-click to open and enlarge the image. The unique file name should have a signature sample ID, sample name, date of birth, sex, and weight.

3. Open Analyze 14.0 on the Desktop and click ***New Workspace***. Select the ***Input/Output*** function on the right-hand side. The user must manually search for the corresponding file opened previously in the Quantum GX2 Database based on the file ID number. The .*VOX file* should be chosen for file type. Then, select ***Calculate it***.

4. Once the image appears in the workspace, click ***Load Volume*** in the bottom left-hand corner of the screen. Click ***Exit***, then ***Rename***. The file desired for analysis may then be designated according to the preferred naming system.

5. Select ***Transform*** on the right-hand side to localize the region of interest within the image. Choose ***Spatial Transforms*** and ***Subregion*** function within, then click ***Extract Sub-Volume*** to delete unnecessary regions and save computing power. Click ***Save Volume*** in the lower left-hand corner of the screen, which saves the transformed image in the new workspace.

6. Select ***Process*** from the right-hand side of the screen. Click ***Spatial Filter*** for the *Process Type*, ***Median*** for the *Filter Type*, ***Kernel Size 3*** for the *Subtype*, and ***Automatic*** for the *Preview Type*. Then, click ***Process Volume*** to generate a smoother image and reduce background noise, overall increasing image quality. Press ***Save Volume***.

**Pause Point:** At this point, three images are saved in the workspace: the original image from the hard drive import, the image after applying the *Transform* function, and the image after applying the *Process* function.

7. Select ***Segment*** on the right-hand side. With the five standards in view, select ***Fabricate Shapes*** under ***Manual***. For dimensions, click ***3-D***, and for *Shape*, click ***Cylinder*** in the *Object to Create* tab, then check the box adjacent to *Filled*.

8. Adjust the radius of the cylinder within the standard to accurately sample the density of the standard. Adjustments of X, Y, and Z coordinates can be made through settings in the *Location* and *Size* section. When the desired size and location adjustments are complete, select ***Apply*** to fix the volume and placement of the cylinder.

9. Click ***Add Object*** and drag the new Object from the original segment to a new standard. Once in position, click *Apply.* Repeat this step so all standards contain a cylinder (Figure S4B, C).

10. Choose ***Save Object*** and open ***Measure*** on the right-hand side.

11. All five circular density samples should be visible within the confines of each standard. Select ***3D*** under the *Sample Type*, then choose ***Enabled Objects***. Under the *Stats to View* section, click ***Size Intensity*** and pick ***Mean***. Check the box for *auto log stats*, and under *Sample Options*, click ***Sample Enabled Objects***. The mean value is generated from all five standards, visible below the scanned image.

12. Open a new Excel file spreadsheet and copy the computed values into the Excel file. Title one column *Object Name* and input the standard densities in this row. Create an adjacent column for *Mean* and then plot the standard curve with the standards on the X-axis and means on the Y-axis. Use Excel to calculate the equation of the standard curve. The trendline of this graph can be referenced when prompted for scaling parameters (“SigmaCT” = slope, “BetaCT” = offset) (Figure S4D).

**C. Quantification of bone mineral density (BMD) of mouse femur**

Upload the mouse data and start the software applications in the same manner as outlined in section B.

**Figure 2.**
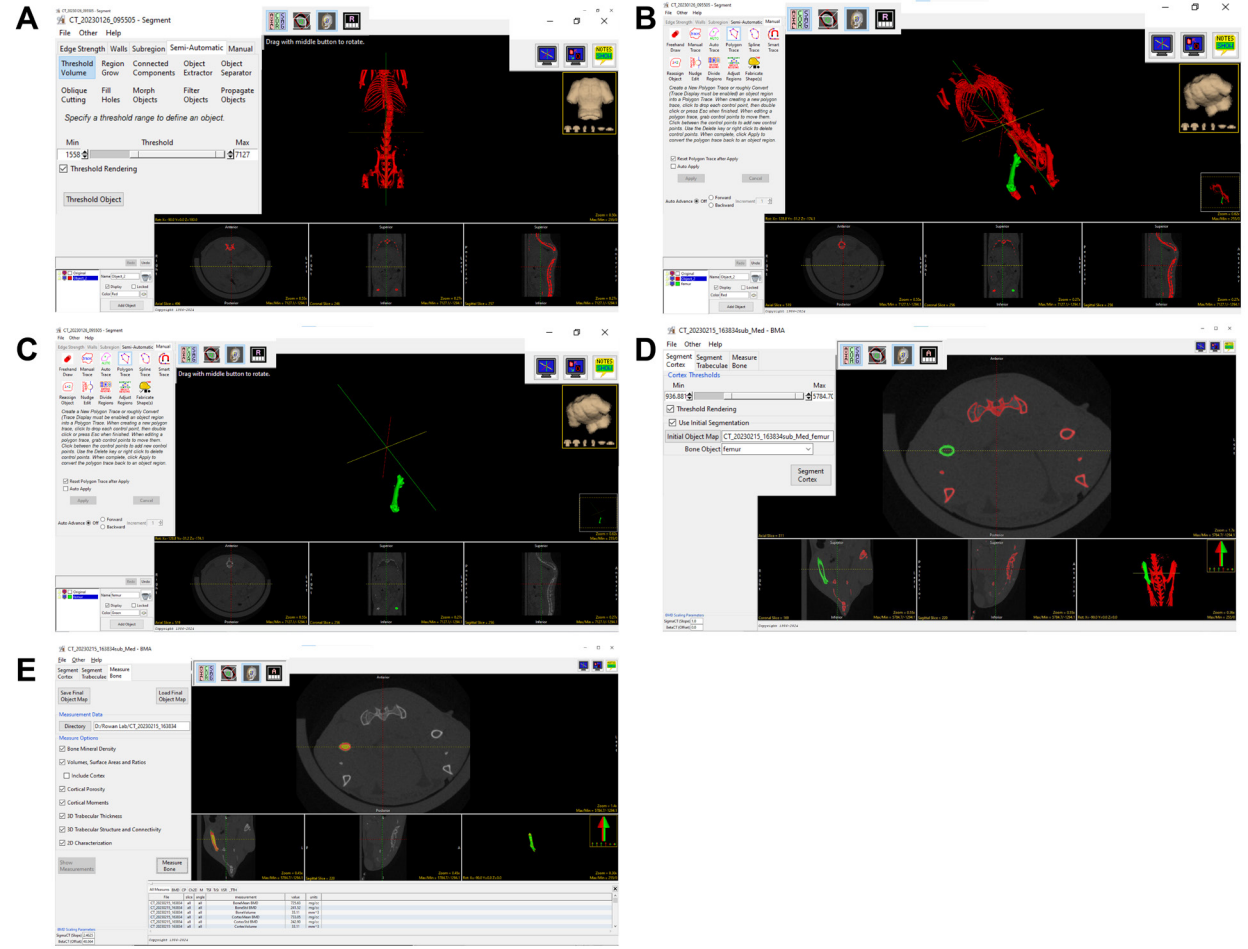
Segmentation of the mouse bone (Femur) and bone mineral density (BMD) quantification. A) Image shows the threshold to segment the mouse skeleton, B) Image showing that the manual trace tool was used to select the femur (green), C) Image shows the removal of other bones with only the femur present, D) This image shows the use BMA tool to segment cortical and trabecular bone E) This image shows the window for calculating and analyzing the density of the femur.

1. Select an image file for loading, then double-click to open and enlarge the image. All captured images should have a unique file name including a signature sample ID, sample name, date of birth, sex, and weight.

2. Open Analyze 14.0 on the Desktop and click ***New Workspace***. Select ***Input*** on the right-hand side. Manually search for the identical sample ID number.

3. Find the corresponding file opened previously in the Quantum GX2 Database based on the file ID number. The .*VOX file* should be selected for file type. Then, click ***Calculate it***.

4. Once the image appears in the designated workspace, click ***Load Volume*** in the bottom left-hand corner of the screen. Click ***Exit***, then ***Rename*** (if desired).

5. Select ***Transform*** on the right-hand side to localize the region of interest within the image. Choose ***Spatial Transforms*** and the ***Subregion*** function within, then click ***Extract Sub-Volume*** to compute the new volume. Click ***Save Volume*** in the lower left-hand corner of the screen.

6. Select ***Process*** from the right-hand side of the screen. Click ***Spatial Filter*** for the “Process Type,” ***Median*** for the “Filter Type,” ***Kernel Size 3*** for the “Subtype,” and ***Automatic*** for the “Preview Type.” Then, click ***Process Volume***. Press ***Save Volume***.

7. Select ***Segment*** on the right-hand side. Under the *Semi-Automatic* tab, click ***Threshold Volume*** and adjust the threshold to find the best range for the object density imaged. Once the optimal threshold range is attained, choose ***Threshold Object*** to isolate the bones (Figure 2A).

8. Select ***Manual***, which enables manual tracing of the desired Object (bone). Press ***Add Object*** (green), and then trace around excess structures outside the object of interest with the cursor to isolate the precise structure (Figure 2B). Objects may be hidden by unchecking the corresponding box. New objects can be added as many times as necessary to refine the specificity of the structure and reduce spatial background noise. Once background noise is traced, these features may be deleted by deleting the “Object” with which they are affiliated. The desired object should be the only “Object” (all others deleted) (Figure 2C) at the end of this process, at which point one must click ***File*** → ***Save Object Map*** → ***Current Directory***.

9. Click ***BMA*** on the right-hand side of the main panel. Select the Excel file in the folder to open the pre-generated phantom plot. Input the SigmaCT (slope) and BetaCT (offset) into the BMD scaling parameters (Figure 2D).

10. Choose ***Initial Segmentation***, click ***Initial Object Map***, and find the saved file of the desired object. Select this file under the Bone Object drop-down menu. *Note: This should be the only object available for input since it is the only one saved.* Click on ***Segment Cortex*** and ***Segment Trabeculae***. Select ***Measure Bone***, utilizing all default settings for this action (Figure 2E).

11. With the cursor, click and drag the image planes along the longitudinal axis of the body to grossly assess the anatomical orientation of the cortex and trabeculae by ensuring the corresponding colors for each respective bone type overlap with the correct area on the image consistently. If the color borders are inaccurate, the *Cortex Threshold* or *Trabecular Threshold* may be adjusted until proper boundaries are accomplished (Figure 2E).

12. Check the *All Measures* tab, which shows numerical values for all structural metrics. These measurements are automatically saved in the .CSV file within the Current Directory (Figure 2E).

**D. Micro-CT quantification of lean tissue (muscle) and adipose tissue**

1. Upload the mouse data and start the software applications in the same manner as outlined in section B.

2. Select an image file for loading, then double-click to open and enlarge the image. All captured images should have a unique file name including a signature sample ID, sample name, date of birth, sex, and weight.

3. Open Analyze 14.0 on the Desktop and click ***New Workspace***. Select ***Input*** on the right-hand side. Manually search for the identical sample ID number.

4. Find the corresponding file opened previously in the Quantum GX2 Database based on the file ID number. The .*VOX file* should be selected for file type. Then, click ***Calculate it***.

5. Once the image appears in the designated workspace, click ***Load Volume*** in the bottom left-hand corner of the screen. Click ***Exit***, then ***Rename*** (if desired).

6. Select ***Transform*** on the right-hand side to localize the region of interest within the image. Choose ***Spatial Transforms*** and the ***Subregion*** function within, then click ***Extract Sub-Volume*** to compute the new volume. Click ***Save Volume*** in the lower left-hand corner of the screen.

7. Select ***Process*** from the right-hand side of the screen. Click ***Spatial Filter*** for the *Process Type*, ***Median*** for the *Filter Type*, ***Kernel Size 3*** for the *Subtype*, and ***Automatic*** for the *Preview Type*. Then, click ***Process Volume***. Press ***Save Volume***.

8. Select ***Segment***. Under the *Semi-Automatic* tab, click ***Threshold Volume***. Adjust the upper and lower limits of the Threshold range until only the well-circumscribed hypodensity regions of the 3D scans are within range (depicted in red).

*Note: This may be verified further by literature discussing appropriate numerical HU values for adipose density.*

**Figure 3.**
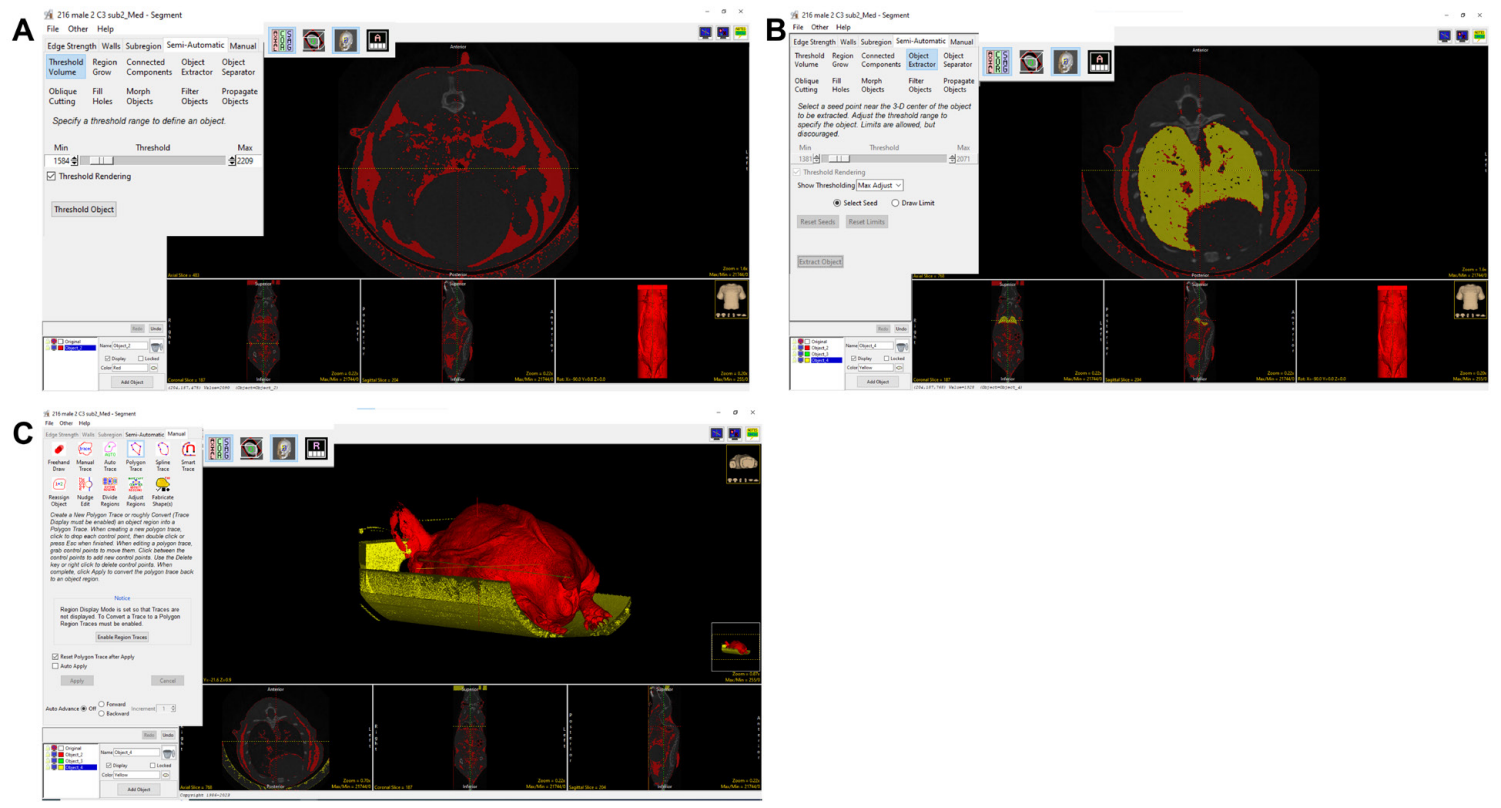
Adipose tissue segmentation and quantification. The 3D volume rendering screenshot shows that the object extractor and threshold volume tools were used to separate fat mass. A–C. Process of separating adipose: A) threshold to segment adipose tissue; B) use ***Object Extractor*** to exclude the lungs; C) use ***Manual Trace*** tool to remove the sample bed.

9. Move the cursor along one of the scans to shift the positional viewpoint within the axial, coronal, and sagittal sections. Ensure that the *Threshold Rendering* function is checked. Then, click ***Threshold Object*** (Figure 3A).

10. Noise can be removed by choosing ***Add Object***. Click the *Manual* tab, select ***Manual Trace***, and trace along the scan to encircle background noise within the new object domains to delineate between the body of the mouse and external structures of similar density detected by the software.

a. **For example:** The lung has a similar HU mean as adipose tissue. To exclude the lung from adipose, from semi-automatic, select ***Object Extractor***, and ***Select Seed*** in the lung region. Adjust the threshold so that the whole lung is automatically selected by the Object Extractor. Select ***Extract Object*** (Figure 3B) to assign the lung to the new Object.

b. To exclude the mouse holder that has a similar HU mean as adipose tissue, go to *Manual*, select ***Polygon Trace*** or ***Manual Trace***, and manually select the mouse holder to a new object (Figure 3C). Once all noise is included in the new *Object*, delete this *Object* by clicking the corresponding box and trashcan icon [Figure 3C (see the inset in the white box)]. Re-title the original Object as *Fat*.

**Figure 4.**
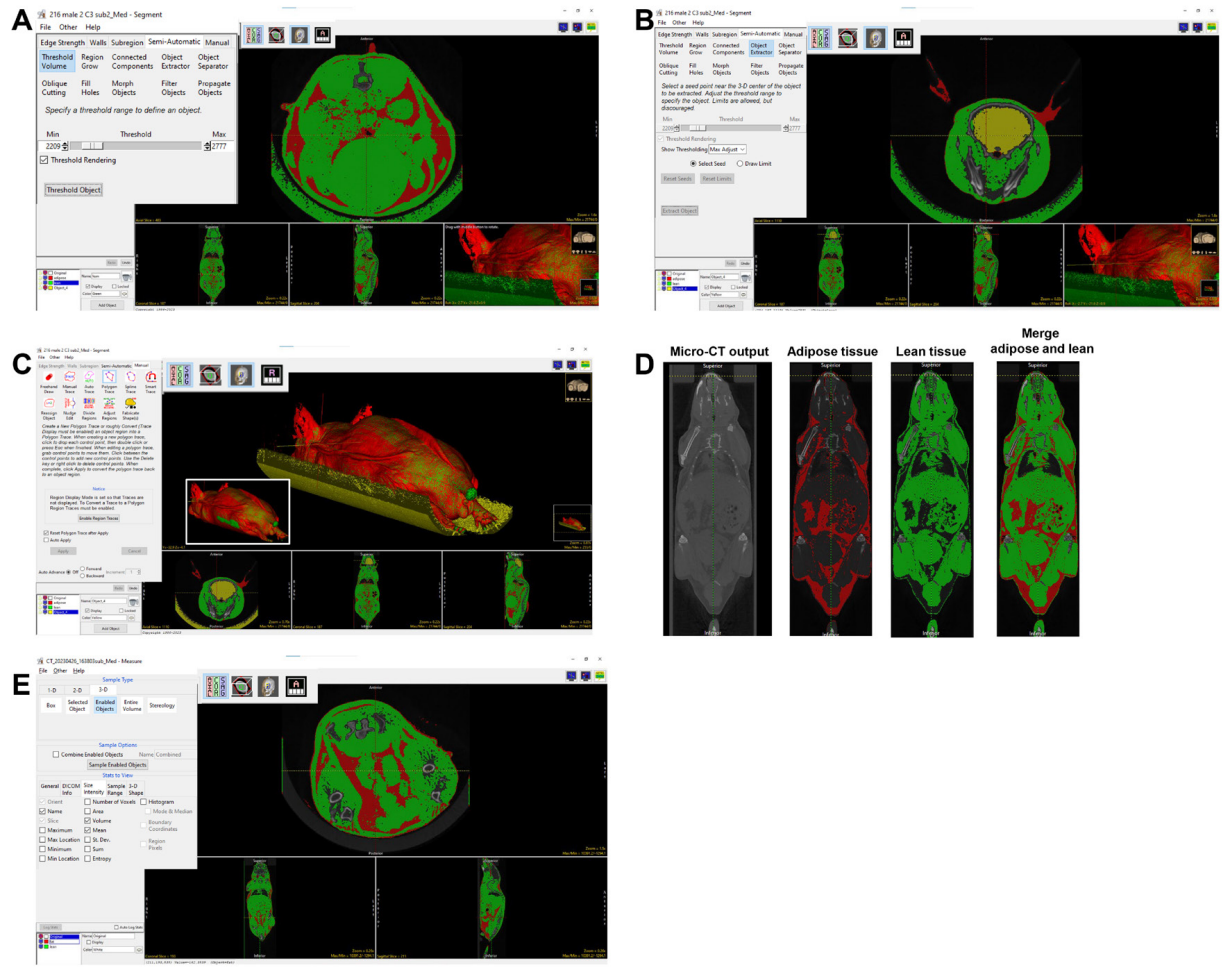
Lean tissue segmentation and quantification. The object extractor and threshold volume tools can be used to separate lean mass, as seen in the 3D volume rendering screenshot. A–C: Process of separating lean tissue: A) threshold to segment lean tissue; B) use Object Extractor to exclude brain; C) use Manual Trace to remove the sample bed; the image in the inset shows the merged 3D rendering of lean and adipose tissue. D) Coronal view of the mouse micro-CT image, segmented adipose tissue, segmented lean tissue, and all three images merged. E) Screenshot of measure tool to quantify the adipose and lean mass volume.

11. To isolate the lean tissue, click ***Add Object*** and adjust ***Threshold*** within ***Threshold Volume*** under the *Semi-Automatic* tab so that the filled regions within the threshold range show color complementary to the fat and exclude bone. Check and uncheck the objects for *fat* and *lean tissue* to assess that the borders of each threshold range are non-overlapping and identifiable. Move the cursor along one of the scans to shift the positional viewpoint within the axial, coronal, and sagittal sections. Ensure that the *Threshold Rendering* function is checked. Then, click ***Threshold Object*** (Figure 4A).

12. Noise can be removed by choosing ***Add Object***. Click the *Manual* tab, select ***Manual Trace***, and trace along the scan to encircle background noise within the new object domains to delineate between the body of the mouse and surrounding pieces of equipment, as well as external structures of similar density detected by the software.

a. **For example:** The brain has a similar HU mean as lean tissue. To exclude the brain from lean tissue, from *semi-automatic*, select ***Object Extractor*** and ***select seed*** in the brain region. Adjust the *Threshold* so that the *Object Extractor* automatically selects the whole brain. Select ***Extract Object*** (Figure 4B) so the brain can be assigned to the new Object.

b. To exclude the mouse holder (which has a HU mean similar to lean tissue), from *Manual*, select ***Polygon Trace*** or ***Manual Trace***, and manually assign the mouse holder to a new object (Figure 4C). Once all noise is included in the new Object, delete this Object by clicking the corresponding box and trashcan icon [Figure 4C (see the inset in the white box)]. Re-title the original Object as *lean tissue*. The merged view of segmented lean and adipose tissue is shown in Figure 4D.

13. Click ***File*** → ***Save Object Map*** to save the object map to the current directory. Click the X in the upper right corner to exit.

14. Click ***Measure*** on the right-hand side.

15. Click ***File*** → ***Load Object Map*** → ***Current Directory*** → select the file just saved and click to open.

16. Under *3-D* tab, click ***Enabled Objects***; under ***Stats to View***, click the *size intensity* tab, and select ***Volume*** without changing other default settings.

17. Click ***Auto Log Stats*** and select ***Sample Enabled Objects*** in the *Sample Options* section (Figure 4E).

18. Copy the volumes generated for both *fat* and *lean tissue* underneath the scanned image and paste them into an Excel sheet for further calculation to obtain the percentage of body fat and lean tissue (see Data analysis).

**E. Micro-CT scanning of the lungs with respiratory gating**

1. Anesthetize the mouse with 2.5% isoflurane in the RAS-4 rodent anesthesia system induction chamber.

*Note: It takes 2 min to fully anesthetize the mice.*

**Critical:** For successful respiratory gating, the time between breaths should be >1,000 ms. Thus, observe the mouse during the anesthesia and ensure that the breathing is stable and not too fast.

2. Place anesthetized mice on the mouse bed and close the imaging system door. Adjust the scan settings in the control window as follows: 90 kV; current: 88 µA; acquisition: 36; recon: 36; scan mode: gating 4 min; display settings: off; X-ray filter: Cu 0.06 + Al 0.5.

**Figure 5.**
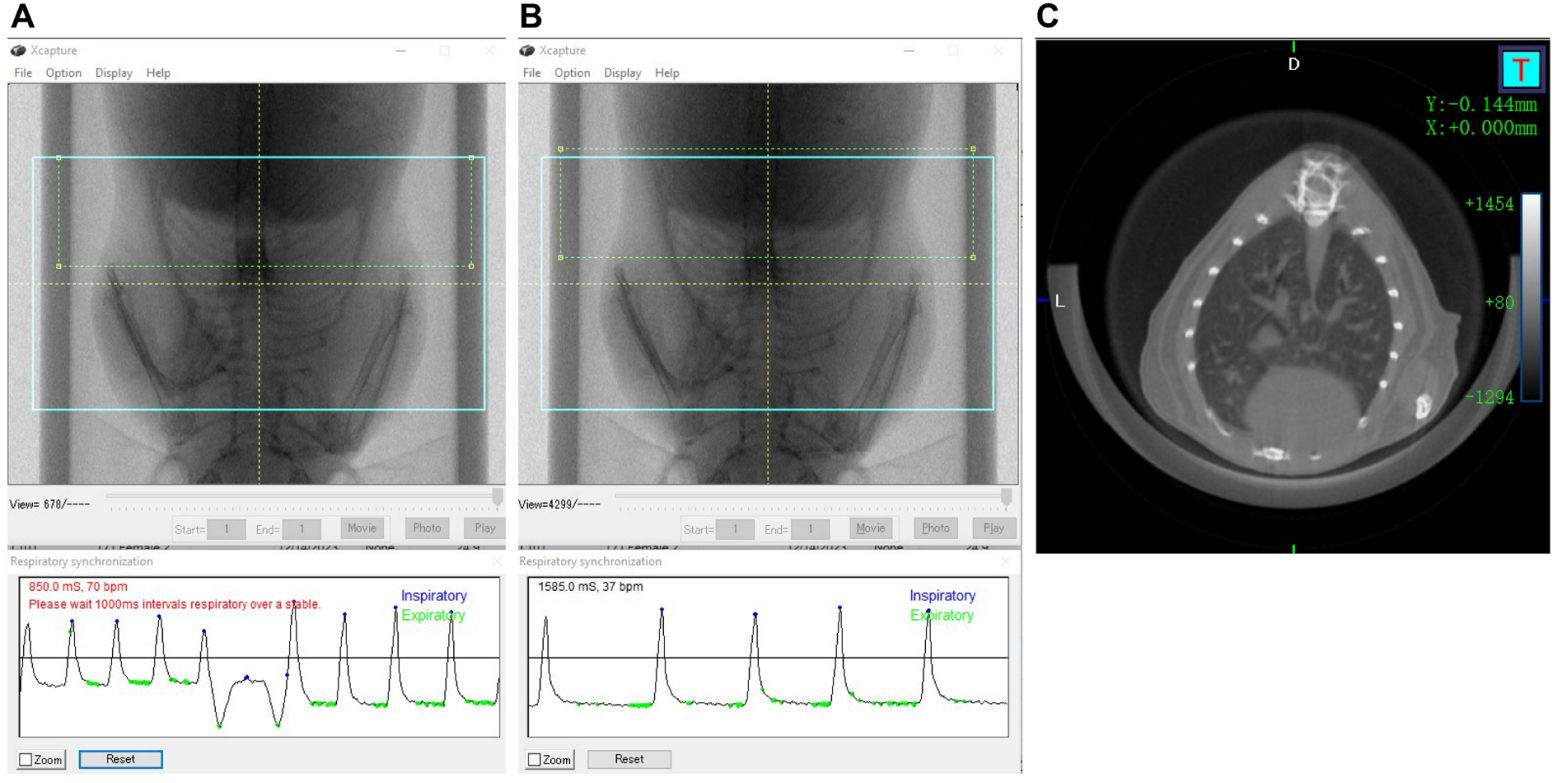
Micro-CT acquisition of mouse lungs. A, B. Video image pop-up screen of the mouse in the Quantum GX2 scanner, with the blue square indicating the FOV for the scan and the green rectangle (dotted) placed partially over the diaphragm for respiratory gating. A) Respiratory interval of less than 1,000 ms, which is incorrect. B) Corrected respiratory interval of more than 1,000 ms. C) Image shows the axial view of the mouse lungs.

*Note: No stitching is necessary in this section because one scan at FOV 36 mm is sufficient to capture the chest area of an adult mouse (Figure 5A).*

3. On the control panel, select the *high speed* scan mode and check the *respiratory gating* icon in the *gating technique* panel.

4. Start ***Live Mode*** and move and resize the green ROI rectangle so that the moving diaphragm is covered by the ROI rectangle. Wait until the breathing is stable and the time between breaths is longer than 1,000 ms (Figure 5B, C).

5. Start CT scan. After the scanning, click ***Reconstruct*** to reconstruct the images of the inspiratory and expiratory phases.

**F. Micro-CT quantification of lung volume and fibrotic structures**

**Figure 6.**
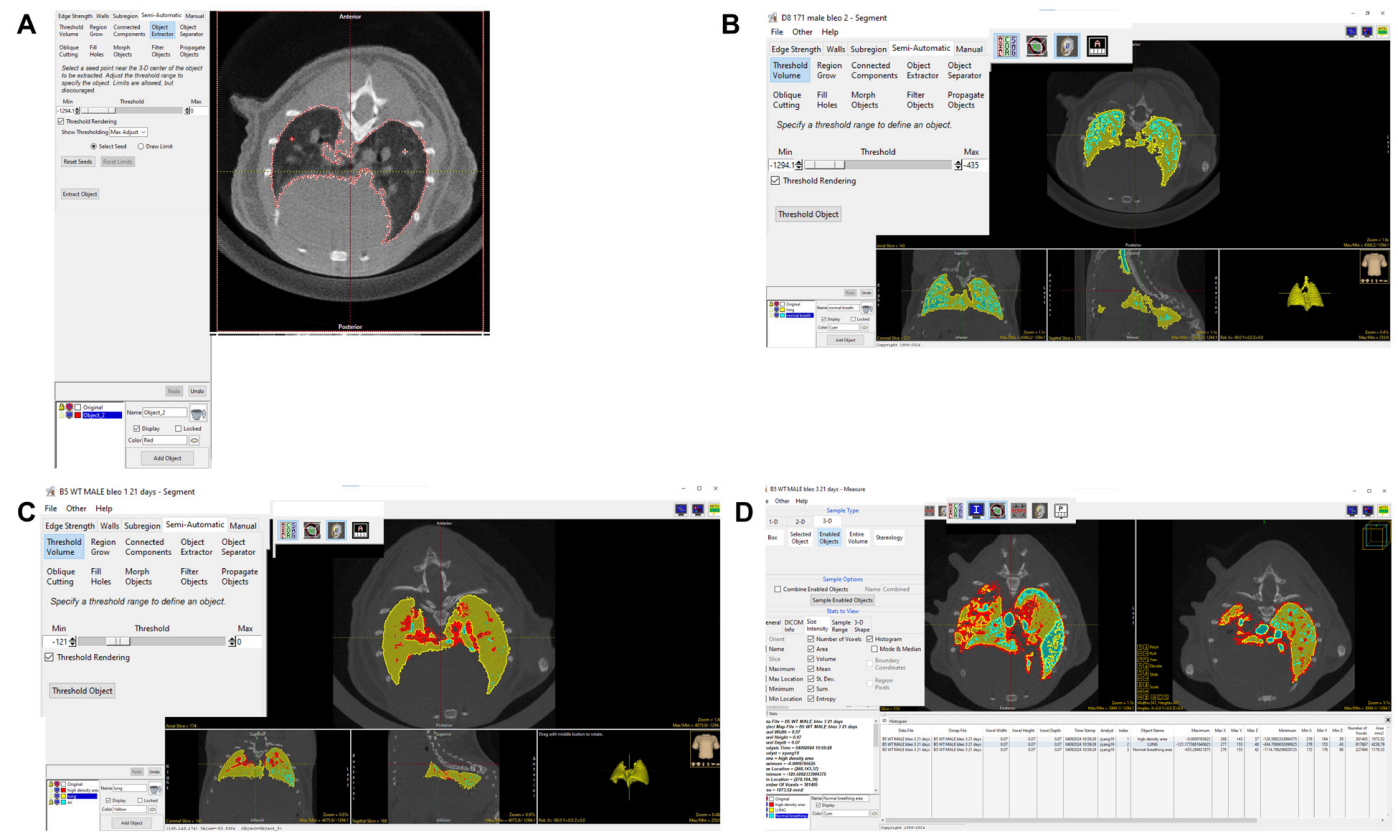
Highlighting and thresholding of lung volume and fibrotic structures using Analyze software. The red areas represent non-aerated regions, indicative of fibrotic tissue. The blue areas represent normally aerated regions, reflecting healthy, well-ventilated lungs. The yellow areas correspond to hypo-aerated regions, reflecting moderate fibrosis. A) This screenshot shows how to draw ROI around the fibrosis area using the *object extractor* tool and adjust the binary threshold values to display the lung with a red outline. B, C) Use the *Threshold Volume* tool to segment normal breath and high-density areas. The blue region is the high-aerated lung area, and the red region is the high-density area in the lungs. D) Image shows that the *Measure* tool was used to calculate the volume of each object.

1. In the Quantum GX2 database application, select the image file to be loaded, then double-click to open and enlarge the image. All captured images should have a signature sample ID, sample name, date of birth, gender, and weight.

2. Launch the Analyze 14.0 application on the desktop and click ***Create New Workspace***. Select the *input* function on the right. Users must manually search for the same sample ID number.

3. Find the corresponding file opened before in the Quantum GX2 Database. Then click the .VOX file to ascertain file type and select the ***Calculate It*** button.

4. After the image appears in the specified workspace, click ***Load Volume*** in the lower-left corner of the screen. Click ***Exit***, then click ***Rename***. Then, specify the file that needs analysis according to the preferred naming system.

5. To crop the image, use the ***Transform*** tab and click ***Subregion***, then click on ***Extract Subvolume*** to remove the excess parts and retain only the section with the lung image. Afterward, click ***Save Volume***, rename the file, and add “_transform” to it.

6. Select ***Segment*** and click the *Semi-Automatic* tab. Select ***Object Extractor*** and ***select seed*** in the lung region. Adjust the *Threshold* so that the *Object Extractor* automatically selects the whole lung. Simultaneously, one can manually input threshold values to adjust the selection range here, using HU values from -1294.1 to 0 (13) as the criteria for delineating the lung area (depicted in red) (Figure 6A).

7. Lock the original image, select the *semi-automatic* button, use the ***Threshold Volume*** key to set the threshold from -1294.1 to -435, and frame the normal breathing image (depicted in blue) (Figure 6B).

8. Continue to select the *semi-automatic* button, use the ***Threshold Volume*** key to set the Threshold from -121 to 0, and frame the high-density area (depicted in red) (Figure 6C).

9. Click ***File*** → ***Save Object Map*** to save the object map to the current directory. Click the X in the upper right corner to exit.

10. Click ***Measure*** on the right-hand side.

11. Click ***File*** → ***Load Object Map*** → ***Current Directory*** → select the file just saved and click to open.

12. Under the *3-D* tab, click ***Enabled Objects***. Under the ***Stats to View***, click *Size Intensity*, and select options such as ***Volume, Histogram, Mean Sum Entropy Number of Voxels, Std. Dev***, etc.

13. Click ***Auto Log Stats*** and select **Sample Enabled Objects** in the sample options section (Figure 6D).

14. Segmentation validation: Compare the segmented areas (non-aerated, aerated, and moderately aerated regions) with the original micro-CT images (Figure 7A–F).

**Pause point:** Focus on the regions highlighted by the red circles to confirm that the segmented areas correctly represent the corresponding severity of lung fibrosis.

**Figure 7.**
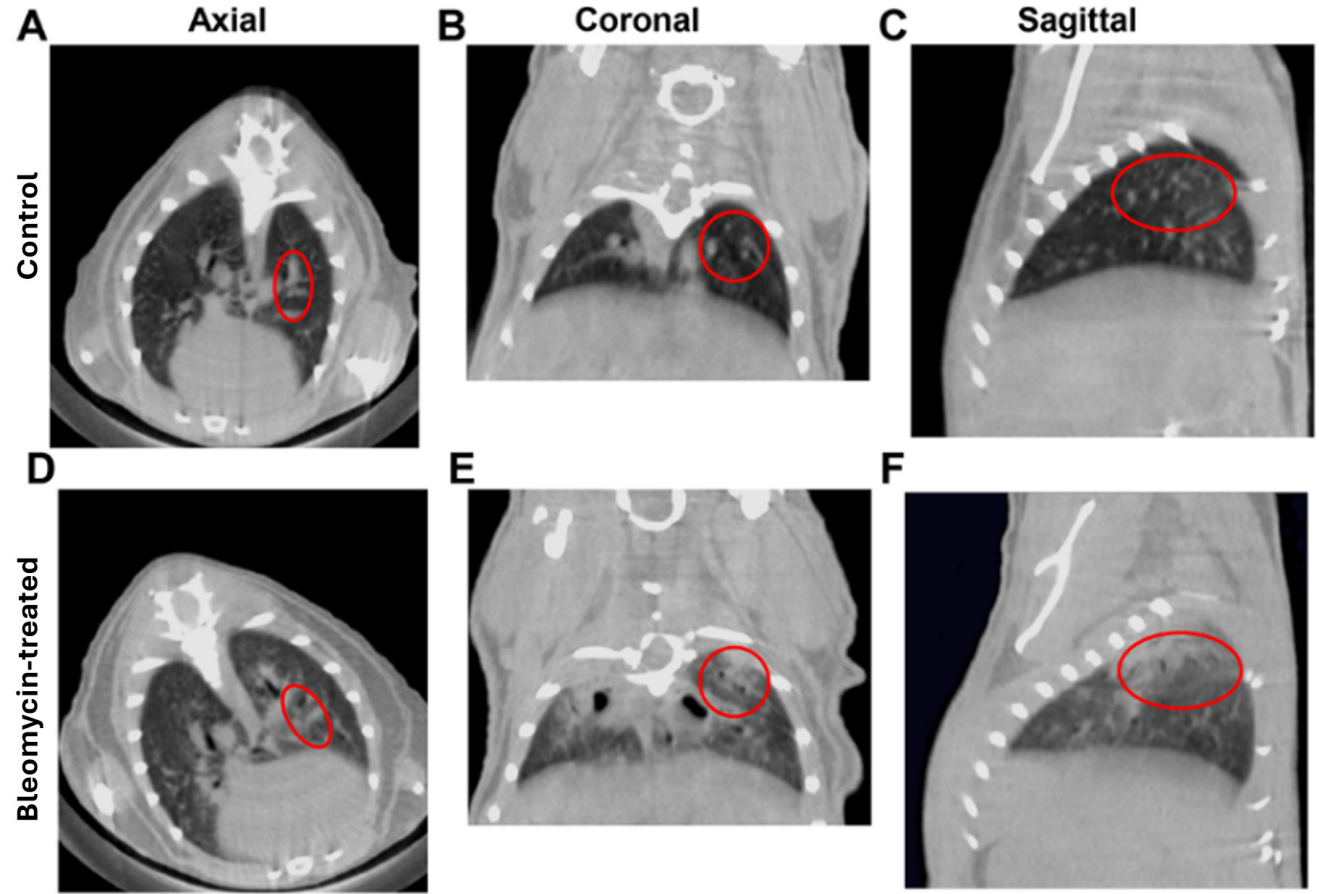
Micro-CT images of normal and fibrotic mice lungs. A–C) Axial, coronal, and sagittal lung CT images of the wild-type control C57BL/6 mouse. D–F) Axial, coronal, and sagittal lung CT images after bleomycin-induced lung fibrosis at day 21. The red circle shows the high-density area of the bleomycin-induced fibrosis mouse lungs and the normal lung area in the control mouse lungs.

## Data analysis

Copy the volume generated for both “fat” and “lean tissue” underneath the scanned image and paste this into an Excel sheet (convert from mm^3^ to cm^3^). Manually input the known densities for each (0.95 g/cm^3^ for soft tissue and 1.06 g/cm^3^ for adipose and lean tissue) [11]. Multiply the volume by density to obtain the mass (g). Then, divide the mass obtained for these values by the body weight to obtain the percentage of body fat and lean tissue.

Data analysis was performed in GraphPad Prism to calculate statistical significance (n = 5–7 biological replicates) using the Student’s unpaired t-test [12]. A p-value of <0.05 was considered statistically significant. Familiarity with GraphPad Prism is necessary.

## Validation of protocol

BMD calculation validation: The BMD values obtained from the calculation of the micro-CT scan of the femur cortical bone of WT mice were between 749.4 and 828.47 HA/cc. These values align with the range previously reported by Mohan et al. (845.4 ± 60.2 HA/cc) [13]. The consistency with previously reported data supports the accuracy of our BMD measurements.

Fat and lean tissue calculation validation: To validate the accuracy of fat and lean tissue quantification, we scanned a WT male mouse using micro-CT and dissected out the abdominal fat and muscle from the hind limbs. The dissected fat and lean tissue were weighed, and the measured weights (fat: 1.169 g, lean: 1.385 g) matched the values calculated from the micro-CT scan (fat: 1.349 g, lean: 1.547 g). The challenges of perfect dissection can explain the differences. This result supports the accuracy of the micro-CT quantification for fat and lean tissue.

Fibrosis scan validation: To validate the accuracy of fibrosis quantification, we performed a micro-CT scan and H&E staining on the same lung tissue samples. The micro-CT results showed that mice with lower aerated lung volumes had higher levels of fibrosis, as evidenced by the increased fibrotic areas in the corresponding H&E staining. This correlation between micro-CT and histological analysis supports the accuracy of lung fibrosis measurement using micro-CT.

## General notes and troubleshooting

**General notes**

1. **Experimental design.** The Quantum GX2 micro-computed tomography scanner, which is commercially available, is used in this protocol to optimize image acquisition parameters for low radiation dose, high-resolution, and high-throughput computed tomography imaging. This methods paper presents a simplified workflow for acquiring and analyzing micro-CT images for mouse body composition, BMD, and mouse lungs by comparing normal and fibrotic lungs. It takes 4–8 min to acquire an image of each animal and 10–30 min to analyze the data. Researchers should be able to use this approach in various mouse models for research purposes if they have a basic understanding of medical imaging, animal care, and software analysis.

2. **Imaging workflow.** The Quantum GX2 is an advanced micro-CT imaging system (Figure 1A) designed for pre-clinical research across a wide range of applications. The RAS-4 Rodent anesthesia system (Figure 1B) connected to the Quantum GX2, where the animal can be kept anesthetized for several minutes (Figure 1C), is ideal for non-invasive, longitudinal studies, producing high-quality images (Figure 1D). The instrument has four fields of view ranging from 18 to 86 mm, with the highest resolutions of 2.3 µm voxels. Quantum GX2 is ideal for imaging pre-clinical animals, including mice, rats, rabbits, zebrafish, and ex vivo samples. This system is also capable of two-phase retrospective respiratory and cardiac gating.

3. **Radiation dosing.** The X-ray radiation dose is an important consideration in micro-CT application. Although micro-CT provides high-resolution images in a non-invasive manner that can be valuable in longitudinal studies, X-ray exposure can potentially affect both DNA repair and the cell cycle in mice [14]. Depending on the strain, the LD50/30 for mice is approximately 5–7.6 Gy [15]. However, the radiation dosage for a respiratory-gated lung scan is about 926 mGy. Thus, to avoid damage caused by radiation, the mouse should not be scanned too frequently. For reference, the mice utilized in this study were safe at a rate of two scans per month. Parameters in micro-CT scanning influencing X-ray dose include the exposure time, type of filters, voltage, and current. In longitudinal studies, researchers should optimize these parameters to minimize the radiation dose while obtaining high-quality images [14,15].

**Troubleshooting**

Problem 1: The prescribed stepwise quantification does not account for differences in adipose tissue.

Possible Cause: Similar density values between subcutaneous and visceral fat cause a lack of distinction between these tissue subtypes after micro-CT scanning acquisition.

Solution: The only method by which such tissue types may be separated would be through manual segmentation based on known anatomical location. Figure 1 in the work by Judex et al. demonstrates manual segmentation for this purpose [14], which is not discussed in this article.

**Other limitations:**

1. The high cost of micro-CT can limit accessibility, especially for smaller research labs or institutions with limited funding.

2. The learning curve associated with micro-CT technology may lead to inconsistencies in data quality and interpretation, particularly among less experienced users.

3. Without adequate training resources, researchers may struggle to fully utilize micro-CT technology's capabilities or produce reliable, reproducible results. This could lead to discrepancies in data quality.

4. Animal handling and animal positioning could impact the image quality and measurements.

## Supplementary information

The following supporting information can be downloaded here:

1. Figure S1. Gain and HU calibration

2. Figure S2. Nose cone setup for isoflurane anesthesia delivery using the RAS-4 system

3. Figure S3. This screenshot image shows the settings for creating a new Job scan with five stitches

4. Figure S4. BMD calibration using phantom
